# Differential Scanning Calorimetry as a Monitoring Tool for the Effectiveness of Therapeutic Plasma Exchange in Anti-AChR Myasthenia Gravis, Anti-MuSK Myasthenia Gravis, and Myasthenic Syndrome: A Case Series

**DOI:** 10.3390/jcm14144968

**Published:** 2025-07-14

**Authors:** Viktoria Ilieva, Boris Tenchov, Daniela Virovska, Denitsa Nencheva, Maksim Kalayanov, Alexandar Farfarov, Yordanka Yamakova, Silviya Abarova

**Affiliations:** 1 Department of Anesthesiology and Intensive Care, University Hospital “Alexandrovska”, Georgi Sofiiski Str. No. 1, 1000 Sofia, Bulgaria; a.farfarov@medfac.mu-sofia.bg (A.F.); y_yamakova@hotmail.com (Y.Y.); 2 Medical Faculty, Medical University of Sofia, Akad. Ivan Geshov Blvd. No. 15, 1000 Sofia, Bulgaria; btenchov@gmail.com (B.T.); dvirovska@medfac.mu-sofia.bg (D.V.); dtognini@medfac.mu-sofia.bg (D.N.); kalayanovmaksim@gmail.com (M.K.); sabarova@medfac.mu-sofia.bg (S.A.)

**Keywords:** Myasthenia Gravis, myasthenic syndrome, differential scanning calorimetry, therapeutic plasma exchange, protein denaturation

## Abstract

**Background/Objectives:** Myasthenia Gravis (MG) and myasthenic syndrome (MSyn) are neurological disorders induced by different types of autoantibodies, characterized by generalized muscle weakness, sometimes involving the respiratory muscles and necessitating ventilatory support. One therapeutic option for severe Myasthenia Gravis (MG) is total plasma exchange (TPE). This procedure reduces the concentration of autoantibodies by extracting the patient’s plasma and replacing it with donor plasma. The TPE efficacy varies among different types of MG, and patient response to TPE is evaluated solely through clinical markers, such as muscle strength. So far, no laboratory method is available for monitoring TPE treatment progress. **Objective:** In this study, we aimed to determine whether differential scanning calorimetry (DSC) of blood plasma from myasthenic patients is an appropriate tool to monitor and evaluate their condition and the TPE effect. **Methods:** We performed DSC prior to and after TPE course on blood plasma from three patients with different types of MG: Case 1. Patient with Acetylcholine Receptor Myasthenia Gravis (AChR MG); Case 2. Patient with Muscle-specific tyrosine kinase Myasthenia Gravis (MuSK MG); Case 3. Patient with Myasthenic syndrome (MSyn). **Results:** DSC thermogram examination revealed increased plasma protein fractions, primarily immunoglobulins (IG), as well as to some extent fibrinogen, relative to a suppressed serum albumin fraction. Successive TPE procedures resulted in IG fraction decline in AChR MG (Case 1) and MSyn (Case 3), and upsurge of the IG fraction in MuSK MG (Case 2). These findings aligned with the clinical presentation of all three cases. **Conclusions:** DSC revealed distinct, very significant differences in the heat capacity profiles of blood plasma from MG patients relative to healthy controls, as well as strong TPE influence on the plasma thermal behavior. DSC showed promise as a reliable and informative technique for the monitoring of myasthenia and TPE effects across diverse myasthenic patient populations. Further research is needed to confirm and expand on these findings.

## 1. Introduction

Myasthenia Gravis (MG) is an autoimmune neuromuscular disorder characterized by varying degrees of muscle weakness, which can range from isolated ocular involvement to generalized forms, potentially leading to acute respiratory failure [1,2]. The condition is caused by the production of antibodies that disrupt the conduction of nerve signals at the neuromuscular synapse [3]. Three types of antibodies can cause MG: antibodies against the acetylcholine receptors (anti-AchR), muscle-specific kinase (anti-MuSK), and lipoprotein receptor-related protein 4 (anti-LRP 4) [1,2,3,4]. Eighty to ninety percent of MG patients have anti-AchR antibodies. Forty to seventy percent of anti-AchR negative MG patients are positive for anti-MuSK antibodies, and two to fifty percent for anti-LRP 4 antibodies [1,5].

The anti-AchR antibodies belong to the IgG1 and IgG3 subclasses [6], the anti-MuSK antibodies are mainly IgG4 [7], and the anti-LRP4 antibodies are primarily IgG1 [8]. Although each of these antibodies ultimately causes the same disease—disruption of signals at the neuromuscular junction—it is essential to classify them, as they require individual therapeutic strategies.

Patients with AChR MG respond to treatment with pyridostigmine (an acetylcholinesterase inhibitor), corticosteroids, immunoglobulins, and immunosuppressants. In contrast, MuSK MG patients do not respond well to acetylcholinesterase inhibitors and may require higher doses of immunosuppressants [9,10]. Therapeutic plasma exchange (TPE) is a treatment option for myasthenic crises in both AChR MG and MuSK MG [11,12], but its efficacy may vary between these two subtypes [11,12].

Myasthenic syndrome (MSyn) is a related but distinct autoimmune disorder that also affects the neuromuscular junction. In myasthenic syndrome, antibodies different from anti-AChR, anti-MuSK, and anti-LRP4 or a combination of anti-AChR and other antibodies disrupt the conduction of nerve signals at the neuromuscular junction, thereby causing muscle weakness. MSyn is very rare and can be provoked by various entities, some of which are drugs [13]. One class of drugs that can cause MSyn are immune-checkpoint inhibitors, which are used as target therapy for various forms of cancer. The first line therapy for immune checkpoint inhibitor-induced MSyn are corticosteroids but up to 30% of patients do not respond sufficiently and physicians opt for other types of treatment. Current recommendations state that MSyn associated with immune checkpoint inhibitor use should be treated by discontinuation of the causative agent and application of corticosteroids first. If the patient’s condition does not improve, the steroid dose should be increased. If this strategy still does not work, a course of intravenous immunoglobulin and/or TPE (simultaneously with steroids) comes as second line therapy. If the results are insufficient, immunosuppression with rituximab should be applied [14]. Neurologic adverse events, related to immune checkpoint inhibitors, can also be chronic diseases, like the other forms of MG. They can be classified as fulminant (if resulted in death within 12 weeks), monophasic (if resolved within 12 weeks), and chronic (if persisted beyond 12 weeks). Chronic cases can be identified as active (if there is indirect evidence of ongoing inflammation) and inactive (if patients have neurologic sequelae without ongoing inflammation) [15].

In severe MG and MSyn, TPE is used as a rescue therapy. Still, it is difficult to evaluate its effectiveness immediately after the procedure, as patients are often on mechanical ventilation. TPE, also known as plasmapheresis, is a procedure designed to remove the harmful auto-antibodies from circulation. It involves the extraction of the patient’s native plasma through a plasma filter, which is subsequently replaced with donor plasma, albumin, or sterile saline. At the same time, the remaining blood components—primarily red blood cells—are returned to the patient. TPE has demonstrated effectiveness in treating various autoimmune disorders, particularly neuroimmunological conditions such as Guillain–Barré syndrome, Idiopathic Thrombocytopenic Purpura, and Myasthenia Gravis [3,4].

The TPE efficacy varies for different types of MG and for patients with the same type of MG. One option to measure the patients’ progress during a TPE course is to measure the titers of their specific antibodies before and after each TPE session, but this is not feasible because these tests are not readily available, results cannot be obtained in the same day (usual time lag is 14 days in most laboratories), and they impose significant financial burdens on patients. Also, the relationship between antibody fluctuations and clinical improvement during any kind of therapy is weak, and controlled trials have yet to be conducted [14,15,16].

Differential Scanning Calorimetry (DSC) is the preferred method for conducting thermodynamic studies on proteins in solution as it makes it possible to directly assess temperature-induced protein unfolding. DSC records the heat capacity profiles (thermograms) associated with thermal denaturation of biological macromolecules, such as proteins and nucleic acids, thereby offering a direct quantitative measure of their thermal stability. Proteins, if not intrinsically disordered at physiological temperatures, exhibit thermograms characterized by specific melting temperature (Tm) and enthalpy (ΔH, the area under the endothermic curve), which together furnish a fundamental thermodynamic signature for that protein.

**Figure 1:**
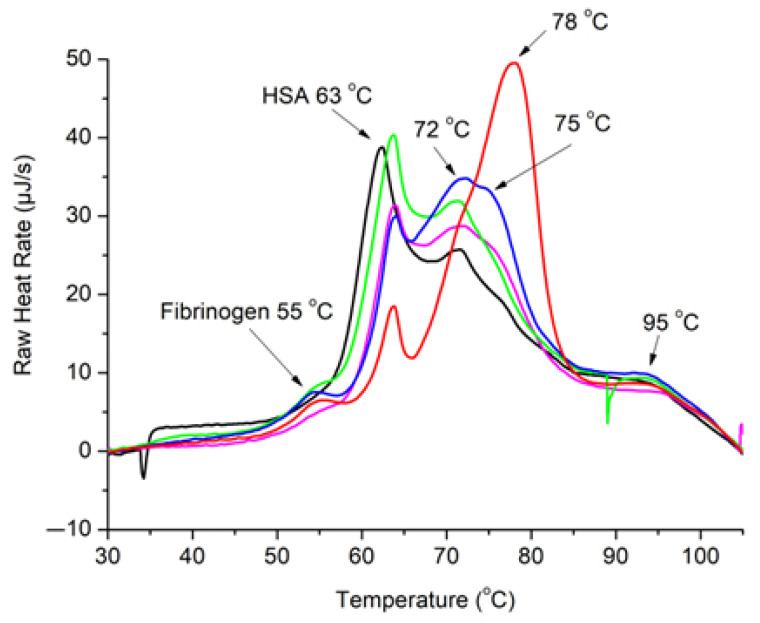
DSC thermograms of blood plasma from a patient diagnosed with AChR MG (Case 1) prior to and after first, second, and third courses of TPE, compared with control thermogram from healthy individuals.

**Figure 2:**
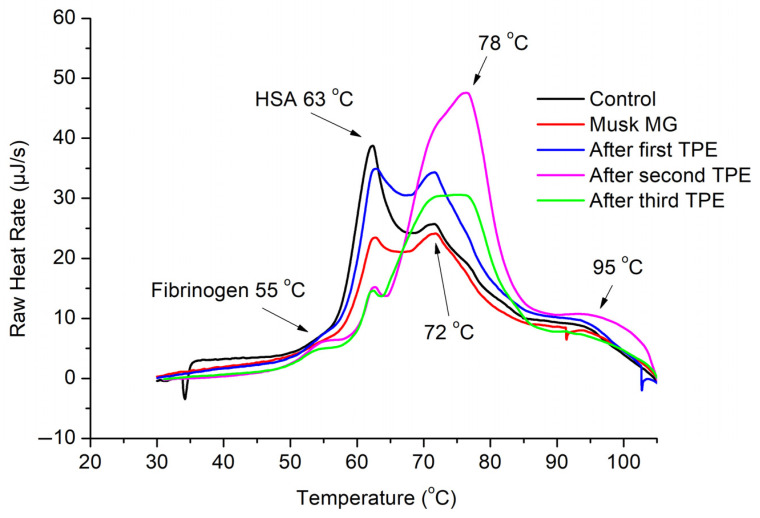
DSC thermograms of blood plasma samples from healthy individuals (Control) and a patient diagnosed with MuSK MG, identified as Case 2, that underwent first, second, and third courses of TPE.

**Figure 3:**
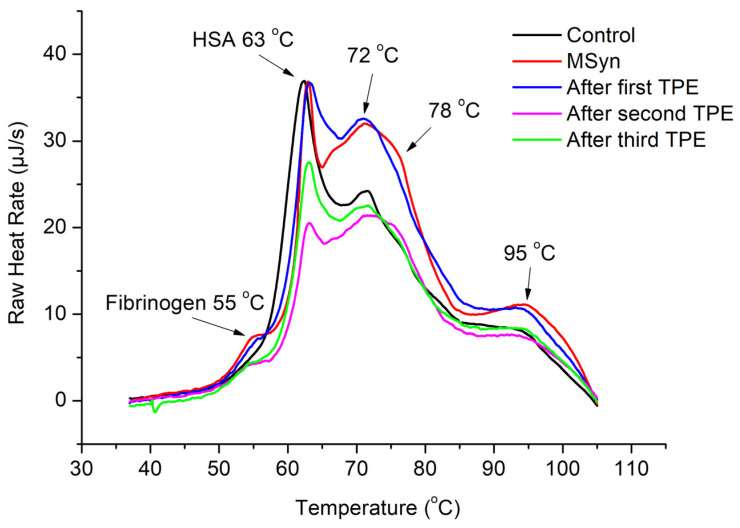
DSC thermograms of blood plasma from Case 3 patient diagnosed with MSyn (myasthenic syndrome) who underwent successive TPE procedures.

Garbet et al. [17] analyzed thermograms from the 16 most abundant blood plasma proteins including albumin, immunoglobulins, fibrinogen, transferrin, and others. Merging together their denaturation profiles according to their weighted ratios in plasma produced the typical DSC pattern of healthy plasma proteome, with dominating peaks at about 63 °C and 72 °C, reflecting denaturation of the most abundant albumin and globulin fractions, respectively, a minor fibrinogen peak around 52 °C, and a hump at about 82 °C usually attributed to IgG and transferrin components (DSC healthy control thermogram obtained in our work is shown in Figure 1, Figure 2 and Figure 3). The calculated curve matched the average experimental thermogram, demonstrating DSC sensitivity to these proteins [18,19].

DSC has been broadly used to detect specific aberrations in the thermal behavior of blood plasma proteins in various diseases, including cervical cancer [20], multiple myeloma [21,22], IgM gammopathies [23], Lyme disease [17,24], chronic obstructive pulmonary disease [25,26], brain tumors, glioblastoma multiforme and low-grade astrocytoma [27], schizophrenia [28], lung cancer [29], breast cancer [30,31], and many others. The DSC profiles could thus provide a novel diagnostic tool for assessing the clinical progression of the disease.

DSC can also differentiate between ligand-bound and unbound states of a protein, as ligands may stabilize or destabilize proteins, thereby affecting their melting temperature (Tm). Research has shown that changes in the thermograms of blood plasma of patients with a particular disease may not be due to changes in the protein concentrations but may rather result from a protein–ligand interaction [17]. If a folded protein binds a ligand, its conformation may be stabilized, leading to increased melting temperature displayed in upward shift of its denaturation peak. Human Serum Albumin (HSA), the dominant protein of blood plasma, is known to bind numerous drugs and their binding may lead to changes in its thermodynamic profile. It is thus possible that alterations in the HSA thermogram due to ligand (drug) binding may alter the overall thermogram of blood plasma [18]. This indicates that DSC is an effective method for observing and analyzing changes in the plasma thermal behavior due to HSA interactions with therapeutic agents and/or disease markers.

Furthermore, given that DSC is a non-invasive and sensitive technique, it is well-suited for monitoring changes in the blood proteome during procedures that modify blood parameters, including TPE. Our previous studies have shown that DSC is effective in assessing the impact of plasmapheresis on healthy individuals and patients with multiple sclerosis [32].

In the present work, we recorded the heat capacity profiles (thermograms) of blood plasma obtained from myasthenic patients with MG and MSyn prior to and after successive TPE treatments. The results revealed very large differences between these profiles and healthy controls.

## 2. Materials and Methods

### 2.1. Study Subjects

This study included three myasthenic patients from the Department of Intensive Treatment at University Hospital Alexandrovska in Sofia. The three patients are labeled as Case 1, Case 2, and Case 3. Their clinical diagnoses were established based on autoantibody concentrations and electromyography. Case 1 was diagnosed with AChR MG; Case 2 had MuSK MG; Case 3 suffered from MSyn in combination with myositis and myocarditis, caused by the immune checkpoint inhibitor pembrolizumab. All three patients had severe MG, with insufficient effect from conservative treatment, and were therefore referred for TPE.

### 2.2. Therapeutic Plasma Exchange

TPE was performed according to the seventh edition of the “Guidelines on the use of Therapeutic Apheresis in Clinical Practice” [33] and the “National Consensus for Intensive Treatment of Diseases by Therapeutic Apheresis in Bulgaria 2014” [34]. The procedure utilized the AcuSmart device for continuous renal replacement therapy and therapeutic apheresis, employing a Plasmart 600 filter. This filter is made from polyether sulfone with a surface area of 0.6 m^2^. Ninety percent of plasma proteins cross through this membrane. Each session had a duration of 2 to 2.5 h, during which 1–1.5 times the volume of the patient’s plasma was separated. The separated plasma was subsequently replaced with 80% donor plasma and the remaining 20% with a 5% albumin solution. Patients were anticoagulated with 1000 UI/h unfractionated heparin.

### 2.3. Preparation of Blood Plasma

Blood samples were collected just before and after the TPE procedures. All participants gave their informed consent. Control samples were taken from eight healthy volunteers, averaging 40  ±  10 years of age. Blood plasma was obtained by centrifugation and stored at −25  °C. Before the DSC measurement, plasma samples were diluted tenfold with phosphate-buffered saline (PBS) at pH 7.4. According to our ELISA determinations, plasma protein concentrations in the diluted samples varied in the range of 5–8 mg/mL, well above the DSC instrument resolution limit of −0.1 mg/mL.

### 2.4. DSC Measurements

DSC was performed using a Nano-DSC microcalorimeter from Thermal Analysis Instruments, which features a measuring cell volume of 300 µL. The reference cell was filled with PBS. The instrument was preheated and calibrated following the manufacturer’s instructions. Two heating scans were conducted at a rate of 1 K/min, spanning a temperature range of 20–110 °C under a pressure of 3 atm. The initial heating scans showed the thermal profiles of the native samples, whereas the subsequent second scans displayed nearly identical profiles typical of denatured samples. To highlight more clearly the thermal transitions in the native samples, the second heating curves of the denatured samples were subtracted from the initial heating scans of the native samples [35].

### 2.5. Monitoring of Clinical Results

In order to monitor the clinical course of the disease during TPE, we evaluated the patients’ Myasthenia Gravis Composite score (MGC score) before treatment initiation and after each TPE session.

## 3. Results

To establish a reference point, control thermograms of plasma samples collected from eight healthy volunteers were recorded. An averaged control shown in Figure 1, Figure 2 and Figure 3 was determined by summing up individual control thermograms and dividing by the volunteer number. The control heat capacity profiles displayed the characteristic DSC patterns of healthy blood plasma, featuring dominant peaks at approximately 63  °C and 72  °C, attributed to denaturation of the prevalent albumin and globulin fractions, respectively. Additionally, a minor fibrinogen peak at approximately 52  °C, as well as a high-temperature hump, were observed.

Controls were compared to thermograms of plasma obtained from myasthenic patients, identified as Cases 1, 2, and 3, prior to and immediately after each of the three TPE procedures performed in all cases. The comparisons revealed remarkably large differences between control and thermograms of plasma obtained from myasthenic patients. Specific details on each case are given below.

### 3.1. Case Report 1

A 48-year-old male diagnosed with AChR MG, who has a history of thymectomy and multiple courses of chemotherapy for thymus carcinoma, presented to the intensive care unit with mild respiratory failure (requiring only oxygen therapy), generalized muscle weakness, and dysarthria, with no other bulbar symptoms. His MSG score was 18. At the time of presentation, he showed no residual tumor mass in the chest. His treatment regimen includes high-dose pyridostigmine and azathioprine. Previously, he was on corticosteroids, but they have since been discontinued. Due to the severity of his condition, we decided to initiate TPE. Three TPE courses were conducted, utilizing a plasma volume exchange fraction of 1.5. After the first TPE procedure, the patients’ MSG score dropped to 12, after the second to 8, and after the third to 2. At the end of the treatment, the patient’s respiratory status improved significantly, and he was able to walk and squat unassisted.

The DSC thermograms we recorded demonstrated a very large divergence between the profiles of healthy controls and those of Case 1 before TPE initiation. The occurrence of AChR MG significantly altered the typical healthy pattern, displayed in a considerable suppression of the albumin peak at 63  °C and rise of a prominent new peak at about 78  °C, which is absent in the control thermograms, along with somewhat elevated fibrinogen peak at about 55 °C (Figure 1).

The presence of a remarkably large new peak at about 78 °C, which dominates the thermogram of blood plasma isolated from the Case 1 patient with AChR MG, and by far exceeds a suppressed albumin peak at 63 °C, appears to be the most important outcome of the DSC measurements in Case 1 illustrated in Figure 1 The origin of the large 78 °C peak cannot be unambiguously determined on basis of the present results. It could be due either to the modified (stabilized) albumin by albumin–drug interactions, or to a strongly enhanced specific globulin fraction. Obviously, further studies would be needed to confirm the properties and clarify the nature of the 78 °C peak.

In another remarkable outcome of the DSC measurements seen in Figure 1, the 78 °C peak was virtually fully abolished by a TPE course. At the same time, the amounts of immunoglobulins produced during the disease gradually declined and approached levels typical of healthy thermograms upon three successive TPE courses. These findings align with the clinical data (note an accompanying MSG score reduction from 18 to 2) and affirm the beneficial effect of TPE treatment.

### 3.2. Case Report 2

A 30-year-old female with MuSK MG presented to our unit with mild respiratory failure, ptosis, dysarthria, mild tongue atrophy, and proximal muscle weakness. Her MSG score was 19, mostly attributed to ocular symptoms and dysarthria. She received treatment with corticosteroids and azathioprine. Upon admission, she was placed on nocturnal non-invasive ventilation, and we initiated a series of therapeutic plasma exchanges (TPEs).

We conducted a total of three cycles of TPE with 1.5 plasma volume exchange. Unfortunately, the patient did not respond to TPE; her muscle weakness persisted and was even exacerbated. After the first TPE procedure, the patient’s MSG score remained at 19, after the second it increased slightly to 21, and after the third TPE it remained at 21. She also experienced symptoms of vertigo. Therefore, we decided to start immunosuppressive treatment with the anti-CD-20 agent rituximab. Several weeks after the application of rituximab, her symptoms gradually improved to an MSG plateau of 6 and she entered remission.

DSC heat capacity profiles of blood plasma isolated from the Case 2 patient with MuSK MG and recorded prior to and after three TPE courses are summarized in Figure 2. Here, again the results show a drastic difference between the thermograms of plasma isolated from the Case 2 patient with MuSK MG and the healthy control (Figure 2).

In alignment with the described above DSC observations on Case 1 patient exhibiting AChR MG, the blood albumin levels were significantly suppressed also in Case 2 patient with MuSK MG. throughout the progression of the treatment procedures. However, the similarity ends here because successive TPE procedures did not improve the thermogram appearance like in Case 1, but rather had the opposite effect and resulted in its deterioration. Notably, following the second and third TPE procedures, the characteristic peaks forming the IG area (approximately 70–80 °C) experienced a dramatic rise, rather than diminishing, indicating an accelerated production of newly synthesized antibodies after the intervention (Figure 2). The DSC findings shown in Figure 2 are well aligned with the clinical data. Note that the MSG score remined almost constant in the range of 19–21 throughout the whole DPE treatment. It is thus clear that TPEs may have different, up to opposite effects in different MG cases, like Case 1 and Case 2 investigated in the present study.

### 3.3. Case Report 3

A 48-year-old male with MSyn after treatment with the immune checkpoint inhibitor pembrolizumab for malignant melanoma, positive for anti-Yo and anti-titin antibodies, was presented to our unit with severe respiratory failure, heart failure (due to myositis, also caused by the pembrolizumab treatment), and generalized muscle weakness. His MSG score at admission was 50. He was intubated and placed on invasive mechanical ventilation. A nasogastric tube was also inserted. We initiated treatment with pyridostigmine and corticosteroids, leading to an improvement in his muscle strength. We successfully weaned him from invasive ventilation, but due to residual respiratory muscle weakness, we decided to place him on intermittent (mainly nocturnal) non-invasive ventilation. After the initial immunosuppressive and anticholinergic treatment, his MSG score reduced to 35. To further accelerate his recovery, we started a course of immunoglobulins; however, the patient did not respond to this treatment and continued to experience resting dyspnea, generalized muscle weakness, dysarthria, and dysphagia. MSG was still 35. Consequently, we opted to perform a series of TPEs and conducted successive TPE procedures with 1.5 plasma volume exchange.

After the first TPE procedure, we observed only a mild clinical improvement with MSG reducing to 32. After the second TPE, he was still bedridden but not so dependent on the non-invasive ventilation with an MSG of 27. After the third TPE procedure, the patient’s muscle strength significantly improved, enabling him to transition to nocturnal non-invasive ventilation only. Also, his neck muscle strength and dysarthria improved, lowering his MSG to 25. To stabilize his condition, he also received two doses of rituximab and was discharged home for further rehabilitation.

Blood samples were collected prior to the TPE treatment and after every TPE procedure. The blood plasma isolated from these samples was examined using DSC. Figure 3 presents a summary of the thermograms obtained about Case 3, who was affected by MSyn, along with control thermogram of healthy plasma from volunteers.

**Figure 4:**
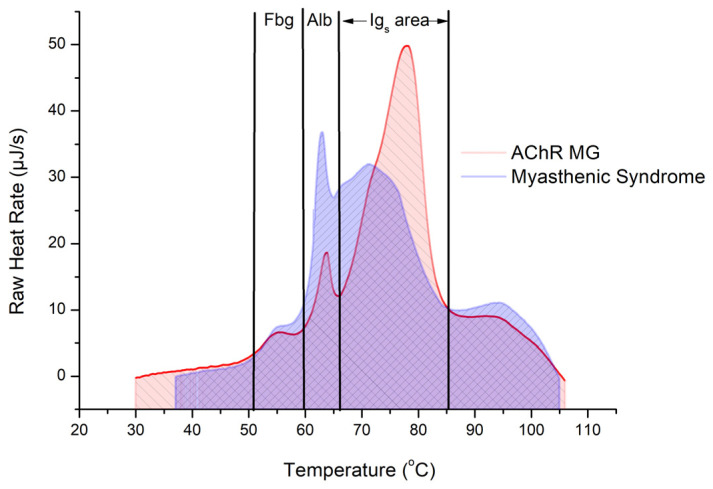
Comparison of the DSC thermograms of Case 1 patient diagnosed with AChR MG and Case 3 patient diagnosed with myasthenic syndrome.

Despite the absence of the disease, some characteristic features of MSyn can be observed in the thermograms of plasma obtained from the Case 3 patient, particularly suppressed fraction of serum albumin, elevated IG area at approximately 70–80 °C, and increased fibrinogen at 55 °C. However, no peak at 78 °C was observed. A comparison of the data from Case 1 with AChR MG and Case 3 with MSyn indicates that the amount of antibodies produced, as given by the area under the curves in the Ig area, appears to be lower in the Case 3 patient with myasthenic syndrome than in the Case 1 patient with AChR MG (Figure 4).

After three TPE courses, both the fibrinogen and immunoglobulin fractions showed a certain decrease, although they did not fully return to control values. This observation suggests that the TPE therapy had a positive impact on plasma proteins, thereby improving the patient’s condition. The DSC findings align with the clinical indicators.

## 4. Discussion

In this work, we used DSC to characterize the denaturational heat capacity profiles of blood plasma isolated from myasthenic patients suffering from AChR MG (Case 1), Musk MG (Case 2), and myasthenic syndrome (Case 3). We also recorded blood plasma thermograms after successive therapeutic plasma exchange (TPE) treatments of the myasthenic patients. The thermograms recorded for myasthenic patients were compared to control thermograms of plasma from healthy individuals. This appears to be the only study so far employing DSC in an investigation on myasthenia and TPE effects.

### 4.1. Synopsis of the DSC Results

A basic, most important result of our study is that DSC revealed drastic differences in the thermal behavior of blood plasma isolated from myasthenic patients and healthy individuals. The large divergences displayed by DSC in the thermograms of myasthenic and healthy blood plasma appear to present a significant advantage and suggest an important argument in favor of using DSC in studies on myasthenia.

DSC revealed remarkably large denaturational peaks in the immunoglobulin (IG) area of blood plasma thermograms of myasthenic patients; in particular, a high peak at 78 °C, which was strongly modulated by repetitive TPE treatments. Other notable effects revealed by DSC include increased plasma protein fractions, primarily immunoglobulins (IG), as well as to some extent also fibrinogen, relative to a suppressed albumin fraction.

To briefly summarize the TPE effects, the DSC results indicated an improvement trend in the blood plasma parameters and gradual approach to a healthy thermogram after successive TPE procedures for the Case 1 patient diagnosed with AChR MG and the Case 3 patient diagnosed with MSyn. However, no improvement but rather a deterioration upon successive TPE procedures was observed for the Case 2 patient diagnosed with MuSK MG. These findings correlate with the clinical data and the status of the patients studied.

### 4.2. Possible Origin of the Notable DSC Peaks in the Immunoglobulin (IG) Area

The notable DSC peaks in the IG area (e.g., in Figure 1 and Figure 2) may actually arise from albumin binding to various pharmaceutical agents such as antibiotics, anticoagulants, pyridostigmine, and corticosteroids. The binding causes the albumin peak to shift to higher temperatures, blending it with the immunoglobulin peaks. As a result, the significant peaks observed before TPE might be due to the formation of albumin–drug complexes, which would enhance the unusual DSC patterns. The DSC data truly offer valuable insights into the thermal behavior of plasma, indicating that protein–drug interactions, rather than just immunoglobulin levels, could play a crucial role in shaping the thermal characteristics of plasma.

### 4.3. Potential Uses of DSC in MG and MSyn

Currently, clinical scales are the gold standard for monitoring the treatment progress of patients with MG during TPE [36]. In addition, two laboratory studies can be conducted—serial AChR-Ab titers or IgG levels [37,38]. If more studies prove the DSC result to be consistent with the clinical course and other laboratory measurements during TPE in MG patients, it can be added as another tool for monitoring patient progress while undergoing blood purification. DSC also has several advantages over the standard test that are used, as follows:

AChR-Ab titers do not always correlate with the severity of the disease course and can therefore be misleading [14,15,16,39]. AChR-Ab titers are more useful in the long-term follow-up of MG patients, as there is a lag between clinical improvement and antibody reduction after plasmapheresis [40]. Also, the typical turnaround time to obtain the results of an AChR-Ab titer test is several days to one week, depending on the laboratory and the specific assay used. For effective monitoring of the progress during TPE, we need a test that can give us results on the same or the next day (DSC results can be obtained roughly 6 h after sample collection). Therefore, AChR-Ab titers are not suitable for real-time monitoring of clinical response during TPE. In addition, non-AChR MG types also exist and measurements of their specific antibodies can take even longer than for AChR-Ab.

In this context, IgG titers seem more feasible [37] because they are unspecific regarding the MG type and results can be obtained several hours after the blood sample was taken. But there are two studies that show the same time lag between IgG reduction in clinical improvement as with AChR-Ab and the magnitude of IgG reduction does not predict the degree of clinical benefit [38,41]. Additionally, IgG measurements are more expensive compared to DSC because DSC does not require specific kits, but only PBS as a dilution solution.

If blood plasma thermal profiles are identified as a useful new measure of antibody titer and a valid tool for monitoring the TPE progress, DSC can be also used in the future in a number of other situations including: 1. Monitoring the efficacy of other types of treatment for MG and MSyn (steroids, immunosuppressants, immunoglobulin); 2. Long-term follow-up of patients on a chronic treatment; 3. Identification of exacerbations of the disease (acute myasthenic crisis); 4. Identification of fulminant, monophasic, chronic active, and chronic inactive types of MSyn and even other types of neurological adverse events associated with immune checkpoint inhibitor use.

### 4.4. Limitations

Our results are based on three case reports only, each one with a different type of MG and therefore different antibody and DSC profile. There is no randomization of the patients because of the small sample size. Herein, we only explore the possibility of DSC to be an appropriate tool for monitoring the state of patients with MG and MSyn, also after the application of TPE. With this research, we set the stepping stone for further studies focusing on the same problem and our group is going to continue collecting DSC data on MG and MSyn patients, as well as on TPE effects, that will be presented in future articles.

Also, we did not conduct a long-term follow-up of the DSC profiles of the patients after the TPE treatment. It is well known that TPE is a rescue therapy and if no immunosuppressive or other disease-specific therapy is implicated, the symptoms will recur because of new antibody production. In the future, we plan to collect samples from patients that have undergone TPE and a course of rituximab or other immunosuppressive therapy in order to explore the possibility of DSC to be useful not only during the acute phase, but also as long-term follow-up laboratory tool.

Considering that DSC profiles originate from various processes associated with disease parameters and the availability of therapy, further research is needed to clarify the effects of plasmapheresis on significant plasma proteins, especially those crucial for Myasthenia Gravis.

## 5. Conclusions

The present work appears to be the first DSC study on myasthenia and TPE effects on myasthenic patients. DSC revealed distinct, very significant differences in the heat capacity profiles of blood plasma isolated from MG patients relative to healthy controls. These differences displayed as increased plasma protein fractions, primarily immunoglobulins (IG), as well as to some extent also fibrinogen, relative to a suppressed serum albumin fraction. A prominent new peak in the IG area was recorded at 78 °C, but was abolished by TPE in a patient with AChR MG. In successive TPE treatments, the IG area in the thermograms of the AChR MG and MSyn patients declined and approached the levels of the controls. However, no such positive effect but rather a deterioration accompanied successive TPE procedures performed on a Case 2 patient diagnosed with MuSK MG.

Our findings suggest that DSC may serve as a reliable and informative method for monitoring and evaluation of myasthenia and TPE effects on patients with MG and MSyn. To confirm and further develop these results, additional research involving a larger patient cohort is essential.

## Data Availability

Data are available upon request from the corresponding author.

## Abbreviations

The following abbreviations are used in this manuscript:

MG	Myasthenia gravis
MSyn	Myasthenic syndrome
AChR MG	Myasthenia gravis with antibodies against the acetylcholine receptors
MuSK MG	Myasthenia gravis with antibodies against the muscle-specific kinase
AchR	Acetylcholine receptors
MuSK	Muscle-specific kinase
LRP 4	Lipoprotein receptor-related protein 4
DSC	Differential scanning calorimetry
TPE	Therapeutic plasma exchange
PBS	Phosphate-buffered saline
IgG	Immunoglobulin G
Alb	Albumin
Fbg	Fibrinogen
MS	Multiple Sclerosis
